# Integrated Blood Transcriptome and Multi-Tissue Trace Mineral Analyses of Healthy Stocker Cattle Fed Complexed or Inorganic Trace Mineral Supplement

**DOI:** 10.3390/ani14152186

**Published:** 2024-07-26

**Authors:** Matthew A. Scott, Kelsey M. Harvey, Brandi B. Karisch, Amelia R. Woolums, Rebecca M. Tracy, Jason R. Russell, Chanda L. Engel

**Affiliations:** 1 Veterinary Education, Research, and Outreach Program, Texas A&M University, Canyon, TX 79015, USA; 2 Prairie Research Unit, Mississippi State University, Prairie, MS 39756, USA; 3 Department of Animal and Dairy Sciences, Mississippi State University, Starkville, MS 39762, USA; 4 Department of Pathobiology and Population Medicine, Mississippi State University, Starkville, MS 39762, USA; 5 Zinpro Corporation, Eden Prairie, MN 55344, USA

**Keywords:** beef cattle, trace element, supplementation, nutrition, RNA sequencing, copper, T-cell, immunity, serum, liver

## Abstract

**Simple Summary:**

Supplementing trace minerals is common for managing bovine respiratory disease in developing beef cattle; however, its effects on their immune and metabolic systems are not fully understood. In this study, we evaluated three different mineral supplement programs and assessed their impact on the concentrations of copper, manganese, cobalt, and zinc, along with the whole blood gene expression, in high-risk beef cattle that remained clinically healthy over a 60-day period. Our results demonstrated that one supplement program, which included amino acid (organically) complexed minerals, led to an increase in gene activity related to adaptive immune system function and the metabolism of carbohydrates and fat-soluble vitamins compared to cattle-fed sulfate (inorganically) sourced complexed minerals. Additionally, the cattle given organically sourced minerals resulted in higher liver concentrations of copper at the end of the study compared to cattle given inorganically sourced minerals. This suggests that tailored mineral supplement programs may improve cattle immunity and mineral absorption. Understanding these effects improves how we manage cattle for diseases such as respiratory disease.

**Abstract:**

Supplementing trace minerals is common in managing bovine respiratory disease (BRD) in post-weaned cattle; however, its influence on host immunity and metabolism in high-risk cattle remains unclear. We aimed to assess the impact of three supplementation programs on liver and serum trace element concentrations and blood gene expression. Fifty-six high-risk beef steers were randomly assigned to one of three groups over 60 days: (1) sulfate-sourced Cu, Co, Mn, and Zn (INR), (2) amino acid-complexed Cu, Mn, Co, and Zn (AAC), or (3) AAC plus trace mineral and vitamin drench (COMBO). Serum and liver biopsies for Cu, Co, Mn, and Zn at d0, d28, and d60 were analyzed from cattle free of BRD (n = 9 INR; n = 6 AAC; n = 10 COMBO). Differences and correlations of mineral concentrations were analyzed via generalized linear mixed models and Spearman’s rank coefficients, respectively (*p* < 0.05). Whole blood RNA samples from healthy cattle (n = 4 INR; n = 4 AAC; n = 4 COMBO) at d0, d13, d28, d45, and d60 were sequenced and analyzed for differentially expressed genes (DEGs) via glmmSeq (FDR < 0.05), edgeR (FDR < 0.10), and Trendy (*p* < 0.10). Serum and liver Cu and Co concentrations increased over time in all groups, with higher liver Cu in COMBO (487.985 μg/g) versus AAC (392.043 μg/g) at d60 (*p* = 0.013). Serum and liver Cu concentrations (ρ = 0.579, *p* = 6.59 × 10^−8^) and serum and liver Co concentrations (ρ = 0.466, *p* = 2.80 × 10^−5^) were linearly correlated. Minimal gene expression differences were found between AAC versus COMBO (n = 2 DEGs) and INR versus COMBO (n = 0 DEGs) over time. AAC versus INR revealed 107 DEGs (d13–d60) with increased traits in AAC including metabolism of carbohydrates/fat-soluble vitamins, antigen presentation, ATPase activity, and B- and T-cell activation, while osteoclast differentiation and neutrophil degranulation decreased in AAC compared to INR. Our study identifies gene expression differences in high-risk cattle fed inorganic or amino acid-complexed mineral supplements, revealing adaptive immune and metabolic mechanisms that may be improved by organically sourced supplementation.

## 1. Introduction

Over the past several decades, North American livestock production systems have developed several cattle management tactics and system concepts focused on disease prevention and performance optimization [1,2,3,4,5,6]. While various health and performance obstacles are faced in these systems, particularly in stocker cattle production, bovine respiratory disease (BRD) development remains one of the primary concerns. To date, BRD remains the leading cause of morbidity and mortality in these systems, leading to economic and welfare concerns for producers and consumers [3,4]. Moreover, stocker cattle, or newly weaned cattle placed into feeder systems, are frequently sourced from varying backgrounds and exposed to stressful conditions such as transportation, sale, and commingling with novel pen mates, which has been shown to challenge their immune system and increase their susceptibility and risk of BRD development [4,7,8,9].

To address this risk, trace element supplementation is a leading management tactic for managing BRD within high-risk stocker cattle populations. Specifically, supplied trace elements, including copper, cobalt, manganese, and zinc, may bolster the innate and adaptive immune responses of cattle, maintain cellular homeostasis and metabolic processes, and mitigate the effects of exposure to stress and infectious agents of BRD [10,11,12,13]. Trace element supplementation, in the context of BRD control, involves developing fortified diets or administering products to meet the macro- and micronutrient requirements of cattle. Ultimately, this may be accomplished through a variety of methods, including those of direct strategies, such as oral supplements, injectable formulations, or the inclusion of trace element-enriched feed ingredients, or indirect strategies, such as pasture management/fertilization and rotational grazing. However, beef cattle producers may supply mineral supplements in excess to match the perceived appetite of the animal, with little regard for the animal’s ability to conserve these elements in tissue and fluid [14]. This type of feeding practice may ultimately result in the over-supplementation of trace elements, which may lead to negative economic and environmental impacts for beef cattle producers without changing performance or disease parameters [15,16]. Further, variation and fluctuation in the intake of mineral supplements by grazing cattle decrease the efficiency of this supplementation strategy [17].

It is apparent that these “one-size-fits-all” strategies remain highly variable in terms of efficacy, and the influence that trace element strategies have on the immune and metabolic responses of high-risk stocker cattle remains disputed. We, therefore, sought to explore and compare the effects of three trace element supplement protocols through a multimodal approach. 

## 2. Materials and Methods

### 2.1. Animal Enrollment and Experimental Design

All animal use and procedures were approved by the Mississippi State University Animal Care and Use Committee (IACUC protocol #21-390) and carried out in accordance with relevant IACUC and agency guidelines and regulations. This study was carried out in accordance with Animal Research: Reporting of In Vivo Experiments (ARRIVE) guidelines [18] and was part of a larger study conducted by our group [19]. A total of 56 commercial crossbred beef steers were purchased from a regional order-buyer within Mississippi and housed at the Mississippi State University Prairie Research Unit (Prairie, MS, USA); previous health and management history was unknown with this group, which is a common occurrence in managing high-risk stocker populations in the United States. Upon purchase, the cattle were loaded onto a commercial livestock trailer and transported for approximately 800 km to simulate long-haul transport conditions. Upon facility arrival (d-1), steers were weighed (average: 230.8 kg; s.d.: 13.7 kg) and placed into a single 2-hectare fescue paddock with access to water troughs. Collected weights were used to randomly stratify cattle into one of 7 drylot pens (6 × 12 m; n = 8 per pen); pen groups were then randomly assigned to receive *ad libitum* access to supplements containing one of 3 treatments: (1) sulfate sources of Cu, Co, Mn, and Zn in feed (INR; custom blend manufactured by Ware Milling, Houston, MS, USA; n = 24); (2) organic complexed source of Cu, Mn, Co, and Zn in feed (AAC; Availa-4 (Zinpro Corp, Eden Prairie, MN, USA); n = 16); or (3) organic complexed source of Cu, Mn, Co, and Zn in feed (Availa-4) and administration of a trace mineral drench product (Profusion; Zinpro Corp, Eden Prairie, MN, USA) prior to shipment, at time of pen enrollment (day 0), and ancillary to any morbidity treatment (COMBO; n = 16); drench dosing was performed according to manufacturer’s instruction. Notably, this animal enrollment was imbalanced due to the availability of pen space at the time of study commencement; feed intake and efficiency, weight gain performance, and health parameters were not statistically different across the inorganically and organically fed groups [19]. All mineral supplements provided were formulated to meet the requirements for macrominerals, Se, I, and vitamins in growing cattle [20]. The INR and AAC sources were formulated to provide the same daily amount of Cu, Co, Mn, and Zn based on 7 g/calf daily of the AAC source as recommended by the manufacturer and previous research with these ingredients [21,22]. According to the manufacturer, ProFusion contains Zn amino acid complex, Mn amino acid complex, Cu amino acid complex, ZnCl, alpha-tocopherol acetate, MnSO_4_, CuSO_4_, Co glucoheptonate, and sodium selenite. Each 30 mL dose provided 720 mg Zn, 400 mg Mn, 250 mg Cu, 15 mg Co, 1 mg Se, and 200 IU Vitamin E. At day 0, all cattle were subcutaneously administered a vaccine for bovine herpesvirus-1, bovine viral diarrhea virus types 1 and 2, parainfluenza-3 virus, and bovine respiratory syncytial virus according to label instructions (Pyramid 5; Boehringer Ingelheim, Duluth, GA, USA), injectable doramectin according to label instructions (Dectomax; Zoetis, Parsippany, NJ, USA), and were given identification ear tags and confirmed to be negative for persistent infection with bovine viral diarrhea virus (BVDV) via ear notch antigen capture ELISA. On day 21, steers were re-vaccinated with the previously mentioned vaccine per label instructions. Steers were subsequently rotated between pens every 14 days to account for the potential confounding effects of the pen and were enrolled in this study for a total of 60 days. Weekly samples of supplement offerings and refusals were analyzed for nutrient content by a commercial laboratory to ensure adequate consumption of mineral products (Table 1). Additional information and data regarding feed intake via electronic feed bunk data, nutritional profiles, wet chemistry procedures, and nutrient provisions are found in previous literature [19].

### 2.2. Sample Acquisition and Handling

Blood samples were collected via jugular venipuncture from all steers on days 0, 28, and 60 into commercial blood collection tubes containing silicon EDTA (BD Vacutainer; Franklin Lakes, NJ, USA) for trace mineral analysis. Concurrently with blood sample collection, liver samples were collected from all steers via needle biopsy (Monopty Core Biopsy Instrument; Becton Dickinson, Tempe, AZ, USA) according to procedures described by Arthington and Corah [23]. Liver and blood samples were immediately placed on ice and stored at −80 °C on the same day of collection before being analyzed via inductively coupled plasma mass spectrometry for concentrations of Co, Cu, Mn, and Zn by the Michigan State Veterinary Diagnostic Center for Population and Animal Health (East Lansing, MI, USA), according to Braselton and colleagues [24].

Due to logistical limitations in the availability of materials, we randomly selected four animals per pen (n = 28 individuals) at the start of the study to collect approximately 3 mL of blood into Tempus RNA blood tubes (Applied Biosystems, Foster City, CA, USA) on days 0, 13, 28, 45, and 60. During the study, two animals (ID_5 (pen 3) and ID_8 (pen 6)) were removed due to musculoskeletal injuries unassociated with the project. Upon study conclusion, a total of 130 blood samples (n = 26 individual cattle) across all five time points remained and were utilized for RNA extraction and sequencing. Tempus RNA blood tubes were collected via jugular venipuncture, then handled and cryogenically stored at −80 °C according to the manufacturer’s recommendations.

### 2.3. Statistical Analysis of Trace Mineral Concentrations

Differences in feed and trace element intake across the three groups across time were performed for these healthy cattle in an identical fashion to our previous work [19]. Differences in mineral concentrations from liver and serum samples of all disease-free cattle enrolled in this study were evaluated through generalized linear mixed effect models (GLMMs) estimated via Gaussian distribution and restricted pseudolikelihood with the Kenward–Rodgers adjustment for degrees of freedom; GLMMs were performed with the glmer function in the lme4 package [25] within R v4.1.2. Independent models included mineral concentration (Cu, Co, Mn, and Zn) as the response variable, evaluating group, time, and their interaction as predictor variables, and random intercepts for pen and animal ID. Differences in generalized least-square means of fixed effects (Wald type III) were considered significant with *p* < 0.10. If significant differences were identified, pairwise comparisons were performed with Bonferroni post-hoc analysis testing to control type I error rates, utilizing a selected α level of 0.05. Violin plots were generated with ggplot2 v3.3.6 [26], utilizing the scale_fill_brewer function with a 3-class color-blind-friendly palette.

The strength of linear associations between serum and liver mineral concentrations over all time points were calculated with Spearman’s rank correlation coefficients with the cor.test function in R Stats v4.1.2, utilizing a selected α level of 0.05. Confidence intervals of Spearman’s Rank correlation coefficients were computed with the spearman.ci function in RVAideMemoire v0.9.81.2 (https://www.rdocumentation.org/packages/RVAideMemoire/versions/0.9-81-2 (accessed on 6 March 2023)), utilizing 95% confidence intervals and 1000 bootstrap replications for each correlation analysis. Scatter plots for each correlation analysis were generated with ggplot2 v3.3.6 [26]. All liver and serum mineral concentrations from these disease-free cattle are found in Supplemental File S1.

### 2.4. Whole Blood RNA Isolation, Sequencing, and Bioinformatic Processing

Total RNA was isolated via the Tempus Spin RNA Isolation Kit (Applied Biosystems) according to the manufacturer’s recommendations. Total RNA from each sample was subsequently assessed for RNA concentration and integrity with a Qubit 4.0 Fluorometer (ThermoFisher, Waltham, MA, USA) and an Agilent 4200 Bioanalyzer (Agilent, Santa Clara, CA, USA), respectively; total RNA samples were of high quality (RIN: 5.8–9.6; mean = 8.9, s.d. = 0.5) and concentrations (ng/μL: 50.8–477.0; mean = 200.8, s.d. = 77.7). Notably, one sample (ID40_D60) possessed an RIN below 6.0. Sample quality control, sequencing library preparation, and high-throughput sequencing were performed by the Texas A&M University Institute for Genome Sciences and Society (TIGSS; College Station, TX, USA). Library preparation for mRNA was performed with Illumina Stranded mRNA Prep kits (Illumina, San Diego, CA, USA) according to the manufacturer’s recommendations. Paired-end sequencing for 150 base pair read fragments was performed on an Illumina NovaSeq 6000 analyzer (v1.7+; S4 reagent kit v1.5), where all 130 samples were barcoded and pooled, and pooled libraries were sequenced equally across two flow cell lanes to avoid batch effects related to the sequencing lane. Importantly, for this study, we retained the sequencing data from those cattle that were never diagnosed nor treated for clinical bovine respiratory disease during the 60-day study period (n = 12).

Raw, demultiplexed reads were acquired from the BaseSpace Sequence Hub (Illumina), where their read quality was assessed with FastQC v0.11.9 (https://www.bioinformatics.babraham.ac.uk/projects/fastqc/ (accessed on 10 March 2023)) and MultiQC v1.12 [27]. One sample (ID48_D13) failed to cluster and was subsequently removed from downstream bioinformatic processing. The remaining samples were concatenated by lane, where read pair trimming retained a minimum read length of 28 bases was performed with Trimmomatic v0.39 [28], scanning each read with a 4-base pair sliding window and removing read segments below a minimum base Phred quality score of 20, removing bases off the start and end of each read if below a Phred quality score of 3, and retaining reads with a length greater than or equal to 28 bases. Following a final read quality assessment with FastQC, trimmed reads were mapped and indexed to the bovine reference genome assembly ARS-UCD1.3 using HISAT2 v2.2.1 [29]. Sequence Alignment/Map (SAM) files were converted to Binary Alignment Map (BAM) files prior to transcript assembly with Samtools v1.14 [30]. Transcript assembly and gene-level expression estimation for differential expression analysis were performed with StringTie v2.2.0, as described by Pertea and colleagues [31,32]. Following assembly and transcript expression estimation, the appending of ambiguous gene-level identifications (“MSTRG” tags) was performed with a custom Perl script provided by Pertea (https://gist.github.com/gpertea/b83f1b32435e166afa92a2d388527f4b (accessed on 17 March 2023)). Raw gene-level count matrices for each sample were generated with the Python3 script prepDE (https://ccb.jhu.edu/software/stringtie/dl/prepDE.py3 (accessed on 17 March 2023)), selecting for an average read length (“–l”) of 150 and all other parameters set to default. All metadata for sequenced samples from these disease-free cattle is found in Supplemental File S2. All sequencing data produced in this study are available at the National Center for Biotechnology Information Gene Expression Omnibus (NCBI-GEO) under the accession number GSE225397.

### 2.5. Dataset Processing and Differential Gene Expression Analyses

Similar to the previously described work, raw gene counts were imported into R v4.1.2 for data sparsity filtering and normalization via the Trimmed mean of M-values (TMM) method [33,34,35]. Following normalization, principal component analysis (PCA) was performed to reduce high dimensionality, determine contributing factors driving expressional variance, and associate clinical metadata components with principal components (PCs) of interest via PCAtools v2.10.0 (https://github.com/kevinblighe/PCAtools (accessed on 21 March 2023)). Based on assumed unequal variance across genes, correlation matrix modeling was applied to the normalized data, specifically log2-transforming gene counts after the addition of a (+2) pseudocount to prevent undefinable log-transformations of any zero counts [36,37]. Modeling through PCA was performed through mean-centering and variance-scaling the data and removing the bottom 10% of genes ranked on variance. Multivariate scree plotting visualized the number of PCs to be retained for further analysis, following the elbow method of clustering validation. Spearman’s rank correlations of the retained PCs were calculated to determine the level of association of each PC with metadata components across all samples, specifically for each trace element supplement group (“AAC”, “COMBO”, and “INR”), the slope of weight gain over time (growth rate; “Growth”), and serum mineral concentrations for Co, Cu, Mn, and Zn at days 0 and 28 (“Serum_Co_D.0”, “Serum_Cu_D.0”, etc.). Any correlations having an FDR < 0.10 were considered significant. A PCA biplot was constructed from the first two PCs with significant correlations to supplement groups (PC1 and PC2), where data ellipses were calculated and visualized from multivariate t-distributions, incorporating the 80% confidence levels of expressional t-distribution across each sample. To identify the genes driving the variation found within each PC significantly correlated with supplement groups, a loadings plot was generated with the top/bottom 0.1% of retained variables across each component loading range.

Following multidimensional reduction analyses (PCA), normalized genes underwent differential gene expression analyses to determine differences between the three trace element supplement groups across time in an ANOVA-like approach. The Bioconductor package glmmSeq v0.2.0 (https://github.com/KatrionaGoldmann/glmmSeq (accessed on 23 March 2023)) was utilized for negative binomial mixed effect modeling of gene counts, following tagwise (gene-specific) dispersion estimation of gene counts. Specifically, time and treatment groups were fitted as fixed effects, and pen on day 0 and individual ID were fitted as random effects for linear modeling, where any gene-level comparison between treatment groups having an FDR < 0.05 was considered significantly differentially expressed. Pairwise comparisons between each treatment group at each time point were then conducted within the Bioconductor package edgeR v3.40.2 [38,39]. Within edgeR, TMM-normalized gene counts were fitted under the generalized linear model (GLM) framework and analyzed via quasi-likelihood F-tests (QLF), following common dispersion estimation. Pairwise gene comparisons were considered significant with an FDR < 0.10. The DEGs identified in both glmmSeq and edgeR modeling were compared and matched in Microsoft Excel (Redmond, WA, USA), indicating agreement between the two models. The matching DEGs were then annotated using the National Center for Biotechnology (NCBI) gene database using the bovine and human reference genomes to annotate “LOC” genes when required. Downstream analyses were performed on the resulting shared DEGs at each timepoint.

### 2.6. Time-Series Segmented Regression Gene Expression Analysis

To better utilize the spatial conditions of this study, we conducted a segmented regression analysis of gene expression for each trace element supplement group over time with the Bioconductor package Trendy v1.20.0 [40]. Trendy allows for the fitting of a “piecewise” regression model of serial gene expression data, permitting the determination of expressional directionality and dynamic changes, or breakpoints, of gene expression across time [40,41]. Filtered raw gene counts from each group were fitted and normalized with the Relative Log Expression (RLE) procedure [42,43]. Across all genes, segmented regression models were fit with the function of each time point, configuring each model for a dynamic change in gene expression overtime (“maxK”) of 1, a minimum of 2 samples present within an expressional segment (“minNumInSeg”), and all other parameters set to default. Genes were considered dynamically expressed if they had at least one segment with a regression segment slope of *p* < 0.10 and if their adjusted R^2^ value was above a threshold in which less than 1% of the genes were retained after a permutation procedure described by Bacher and colleagues (https://www.bioconductor.org/packages/devel/bioc/vignettes/Trendy/inst/doc/Trendy_vignette.pdf (accessed on 25 March 2023); the R^2^ value cutoffs selected were 0.340, 0.305, and 0.316 for AAC, COMBO, and INR, respectively. For downstream analyses, dynamic gene expression patterns were assigned to four directional expression groups, assigning genes based on whether they increased (“Up”), decreased (“Down”), stabilized in trended expression after their respective breakpoint (“Stabilized”), or never possessed a breakpoint (i.e., continuous expressional trend) (“None”) [44]. Visual relationships of the genes identified by the top sparse classifiers were performed with UpSetR v1.4.0, utilizing the Intervene Shiny Application [45,46].

### 2.7. Functional Enrichment Analyses

Significant genes identified through comparative and segmented regression analyses were independently evaluated for enriched pathways and Gene Ontology (GO) terms via KOBAS-intelligence v.3.0 [47]. Within KOBAS, we utilized an overrepresentation analysis method employing hypergeometric distribution and Fisher’s exact testing of specific functional enrichments from DEGs, using the GO knowledgebase, Kyoto Encyclopedia of Genes and Genomes, and Reactome databases [48,49,50]. Any functional enrichment with an FDR < 0.05 was considered significant.

## 3. Results

### 3.1. Statistical Analyses of Trace Element Intake and Concentrations

**Figure 1:**
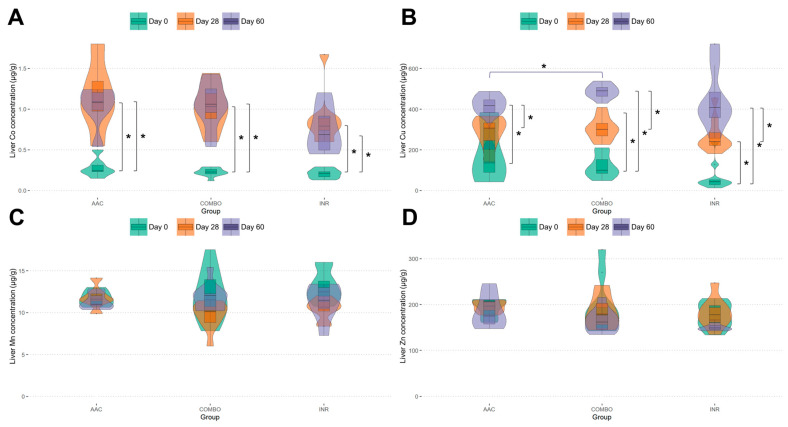
Violin plots of liver concentrations (μg/g) of (**A**) Co, (**B**) Cu, (**C**) Mn, and (**D**) Zn levels. Relative distributions of concentrations are depicted by day (0, 28, 60), median and quartile values are represented by embedded box plots, and any significant differences are described with a star (*).

Table 2 describes differences in feed and mineral intake across time for these three groups of healthy cattle. Specifically, no differences were found in total feed intake; however, Co, Cu, Mn, and Zn were statistically different in AAC cattle when compared to both COMBO and INR cattle. For differences in liver samples, Co concentrations across all three groups significantly increased from day 0 to day 28 and day 60 (*p* < 0.001), with no statistical difference between days 28 and 60 within each group nor between any group at any time point (*p* > 0.05) (Figure 1A). Regarding Cu concentrations, all three groups demonstrated a significant increase in concentrations across each time point (*p* < 0.001), with the only significant difference between groups being a higher concentration in COMBO (500.93 ± 27.31 μg/g) compared to AAC (437.95 ± 39.16 μg/g) at day 60 (*p* = 0.013) (Figure 1B). Regarding Mn and Zn concentrations, no statistical differences were observed between groups at each time point or within each group over time (*p* > 0.05) (Figure 1C,D).

**Figure 2:**
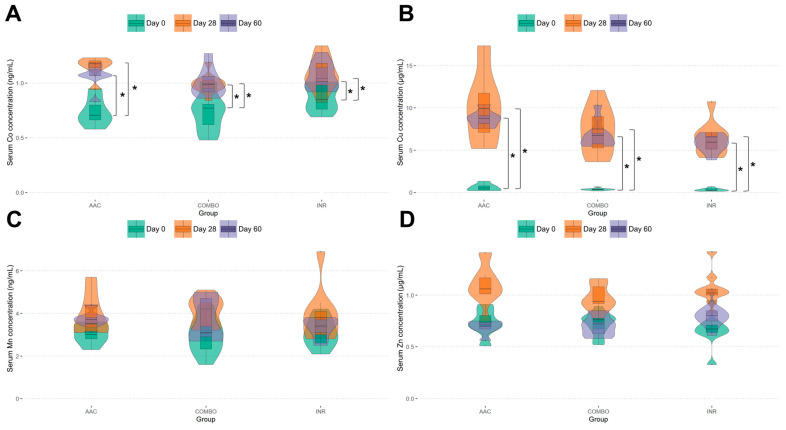
Violin plots of serum concentrations (ng/mL) of (**A**) Co, (**B**) Cu, (**C**) Mn, and (**D**) Zn levels. Relative distributions of concentrations are depicted by day (0, 28, 60), median and quartile values are represented by embedded box plots, and any significant differences are described with a star (*).

For differences in serum samples, Co concentrations were similar in statistical trends to those of liver samples, where all three groups significantly increased Co concentrations from day 0 to day 28 and day 60 (*p* < 0.001), with no statistical difference between days 28 and 60 nor between any group at any time point (*p* > 0.05) (Figure 2A). Regarding Cu concentrations, all three groups significantly increased from day 0 to day 28 and day 60 (*p* < 0.001), with no statistical difference between days 28 and 60 or between any group at any time point (*p* > 0.05) (Figure 2B). Similar to liver samples, no statistical differences were observed with respect to Mn and Zn concentrations between groups at each time point nor within each group over time (*p* > 0.05) (Figure 2C,D).

**Figure 3:**
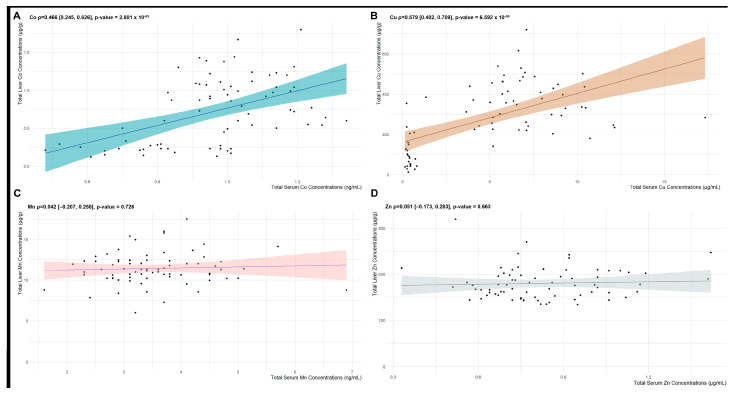
Scatterplot and fitted linear correlation lines between serum (*x*-axis; ng/mL) and liver (*y*-axis; μg/g) concentrations of (**A**) Co, (**B**) Cu, (**C**) Mn, and (**D**) Zn levels.

When comparing the associations between liver and serum mineral concentrations, Co (ρ = 0.466, *p* = 2.801 × 10^−5^; Figure 3A) and Cu (ρ = 0.579, *p* = 6.592 × 10^−8^; Figure 3B) demonstrated significant positive linear associations (*p* < 0.05), whereas no statistical linear association was observed for Mn (*p* = 0.726; Figure 3C) nor Zn (*p* = 0.663; Figure 3D).

### 3.2. Principal Component Analysis

**Figure 4:**
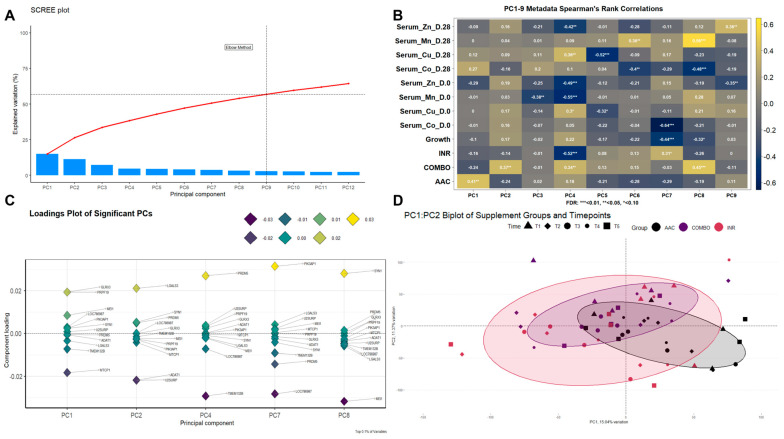
Principal component analysis (PCA) of filtered and normalized gene expression data for all 59 samples. (**A**) Scree plot depicting the first nine principal components (PCs) retained for downstream analyses per the automated elbow method. (**B**) Metadata correlation heatmap with retained PCs via Spearman’s rank correlation coefficients. Color scaling represents the R-value captured between each PC and metadata component; yellow/white cells represent a higher positive value, and purple/black cells represent a lower negative value. Significance was calculated through FDR adjustments and is indicated by * FDR < 0.10, ** FDR < 0.05, or *** FDR < 0.01. (**C**) Loadings plot graph of the top 0.1% annotated genes driving associated variation and directionality (*y*-axis) of PC1, PC2, PC4, PC7, and PC8; genes identified by variance are seen as the most responsible for driving variation with each of the aforementioned PCs. Color (dark yellow to dark purple; positive to negative) demonstrates the corresponding directionality of expression and strength of influence for each gene within each PC. (**D**) Biplot of PC1 and PC2, which possess significant correlation with AAC and COMBO supplementation groups, respectively. Samples were colored by supplementation group (black = AAC, purple = COMBO, red = INR) and shaped by time point (triangle = D0 (T1), diamond = D13 (T2), large circle = D28 (T3), small circle = D45 (T4), square = D60 (T5)). Individual plots (vectors) represent the PC score of the individual sample by gene expression, and vector distances along the *x*- and *y*-axes represent the total variational influence. Ellipses were calculated from multivariate t-distributions and encompassed 80% confidence levels of expressional t-distribution across all time points.

Initial data processing and filtering of the 59 gene-level transcriptomes in this study resulted in 17,716 genes being retained for differential expression and clustering analyses (median library count = 44,216,798 ± 5,441,080). From global gene expression clustering via PCA, the first nine principal components (PCs) were retained for clustering correlation analyses, accounting for 56.84% of the total explained variance within the gene expression dataset (Figure 4A). Following the construction of Spearman’s rank correlation matrix of metadata components with these PCs (Figure 4B), significant correlations were identified with respect to all retained PCs. Growth was correlated with PC7 (3.68% variance explained; r = −0.44, FDR < 0.01) and PC8 (3.18% variance explained; r = −0.32, FDR < 0.10). The AAC group possessed a significant correlation with PC1 (15.04% variance explained; r = 0.41, FDR < 0.05). The COMBO group possessed significant correlations with PC2 (11.37% variance explained; r = 0.37, FDR < 0.05), PC4 (4.72% variance explained; r = 0.34, FDR < 0.05), and PC8 (3.18% variance explained; r = 0.45, FDR < 0.01). The INR group possessed significant correlations with PC4 (r = −0.52, FDR < 0.01) and PC7 (r = 0.31, FDR < 0.10). Serum mineral concentrations from both day 0 and 28 of the study possessed multiple PC correlations; of particular interest was PC4, which was correlated with Cu (r = 0.30, FDR < 0.10), Mn (r = −0.55, FDR < 0.01), and Zn (r = 0.49, FDR < 0.01) at day 0 and Cu (r = −0.42, FDR < 0.05) and Zn (r = 0.36, FDR < 0.05) at day 28. The genes determined to be driving the explained variation within each PC significantly correlated with supplement groups are found in Figure 4C. Genes consistently driving variance for these PCs were *ADAT1*, *GLRX3*, *LGALS3*, *LOC786987*, *MEI1*, *MTCP1*, *PIK3AP1*, *PRDM5*, *PRPF19*, *SYN1*, *TMEM132B*, and *U2SURP*. A biplot of PC1 and PC2 was generated to assess dissimilarities based on the PCs explaining the most variance, with association with the AAC and COMBO groups; the AAC and COMBO samples demonstrated the most dissimilarity, while the INR group appeared to have the highest amount of intragroup variance (Figure 4D).

### 3.3. Differential Gene Expression Analysis

Differential gene expression analysis between the three supplement groups yielded a total of 156 DEGs (Supplemental File S3). When comparing AAC to COMBO, a total of 40 DEGs were identified, with 38 on day 0, one on day 13, and one on day 28. Because day 0 differential gene expression does not represent trace element supplementation and so few DEGs were identified in the subsequent study days, no functional enrichments were identified between AAC and COMBO. When comparing COMBO to INR, eight DEGs were identified, all of which were on day 0; no functional enrichments were found between COMBO and INR. 

**Figure 5:**
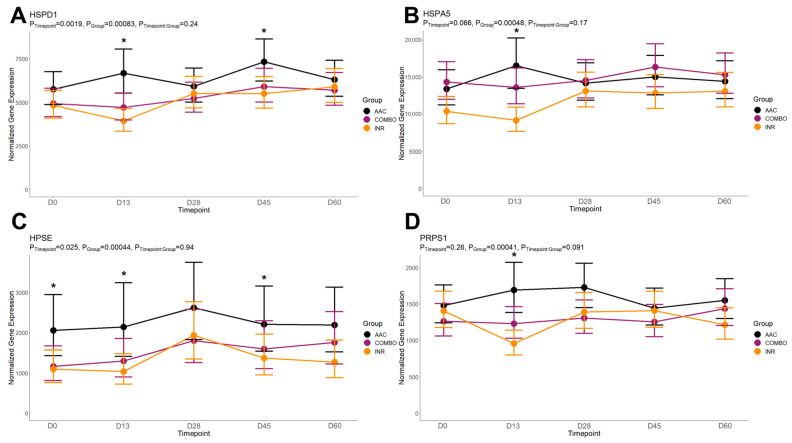
Fitted gene expression modules of genes driving significant functional enrichments between AAC and INR at day 13. Points and bars represent the estimated mean and confidence intervals of normalized expression values for each group within each timepoint, respectively, based on the fitted regression model from glmmSeq. Stars (*) represent significant differential expression based on edgeR analysis (FDR < 0.10). Fitted plots represent the relative expression for (**A**) *HSPD1*, (**B**) *HSPA5*, (**C**) *HPSE*, and (**D**) *PRPS1*.

When comparing AAC to INR, a total of 39 DEGs were identified: one on day 0, 18 on day 13, three on day 28, 16 on day 45, and one on day 60. On day 13, DEGs found between AAC and INR were enriched for 139 GO terms and 47 pathways. Enriched GO terms were primarily related to cell surface activity (increased in AAC), protein binding and transmembrane receptor signaling (increased in AAC), ATPase activity (increased in AAC), neutrophil degranulation (decreased in AAC), B- and T-cell activation/proliferation (increased in AAC), and cellular response to interleukins 4, 7, and 10 (increased in AAC). Enriched pathways were primarily related to carbohydrate metabolism and collagen biosynthesis (increased in AAC), platelet activation and hemostasis (decreased in AAC), metabolism of fat-soluble vitamins (increased in AAC), collagen biosynthesis (increased in AAC), and MHC class I antigen presentation (increased in AAC). The time-course trends of the genes driving several of these functional enrichments are found in Figure 5.

**Figure 6:**
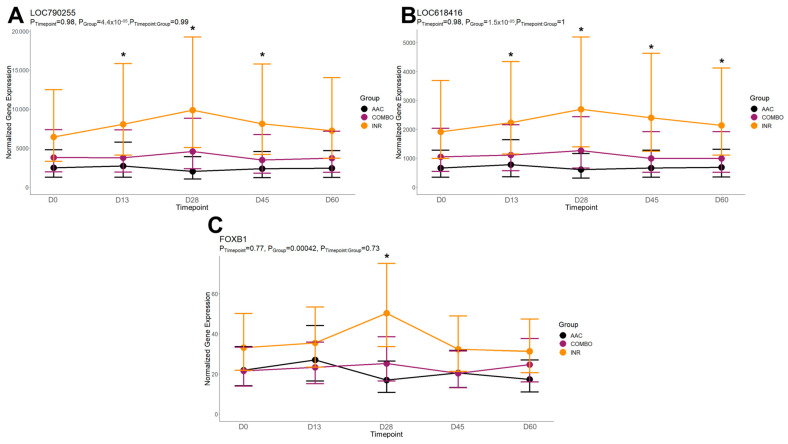
Fitted gene expression modules of genes driving significant functional enrichments between AAC and INR at day 28. Points and bars represent the estimated mean and confidence intervals of normalized expression values for each group within each timepoint, respectively, based on the fitted regression model from glmmSeq. Stars (*) represent significant differential expression based on edgeR analysis (FDR < 0.10). Fitted plots represent the relative expression for (**A**) *LOC790255* (*LILRA6*), (**B**) *LOC618416* (*LILRA6*), and (**C**) *FOXB1*.

On day 28, DEGs were found between AAC and INR enriched for 23 GO terms and two pathways. Enriched GO terms were primarily related to nervous system development (decreased in AAC), neutrophil degranulation and immunological defense response (decreased in AAC), and cellular differentiation (decreased in AAC). Enriched pathways were the B-cell receptor signaling pathway (decreased in AAC) and osteoclast differentiation (decreased in AAC). The time-course trends of the genes driving several of these functional enrichments are found in Figure 6.

**Figure 7:**
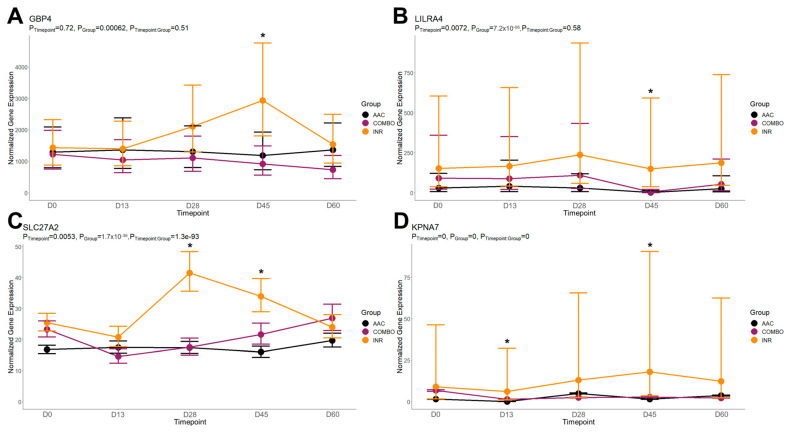
Fitted gene expression modules of genes driving significant functional enrichments between AAC and INR at day 45. Points and bars represent the estimated mean and confidence intervals of normalized expression values for each group within each timepoint, respectively, based on the fitted regression model from glmmSeq. Stars (*) represent significant differential expression based on edgeR analysis (FDR < 0.10). Fitted plots represent the relative expression for (**A**) *GBP4*, (**B**) *LILRA4*, (**C**) *SLC27A2*, and (**D**) *KPNA7*.

On day 45, DEGs found between AAC and INR were enriched for 101 GO terms and 27 pathways. Enriched GO terms were primarily related to neutrophil degranulation (decreased in AAC), negative regulation of interferon-alpha production (decreased in AAC), lipoprotein/lipopolysaccharide binding (decreased in AAC), fatty acid ligase activity (decreased in AAC), B- and T-cell activation (increased in AAC), response to interleukin 7 (increased in AAC), and the positive regulation of interleukins 10 and 12 (increased in AAC). Enriched pathways were primarily related to B-cell receptor signaling (decreased in AAC), interferon signaling (decreased in AAC), bile acid and salt metabolism/synthesis (decreased in AAC), and neutrophil degranulation (decreased in AAC). The time-course trends of the genes driving several of these functional enrichments are found in Figure 7. All functional enrichments found for each comparison are found in Supplemental File S4.

### 3.4. Trend-Wise Gene Expression Analyses

**Figure 8:**
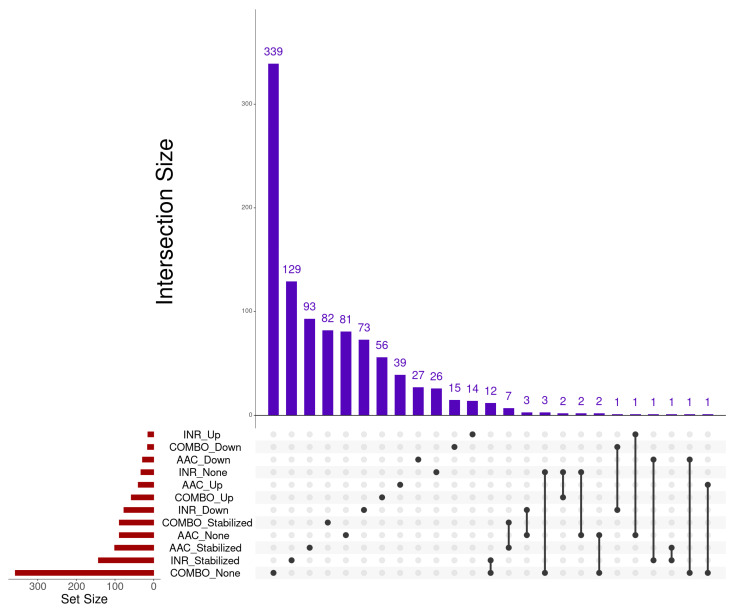
Upset plot indicating the number of overlapping dynamically expressed genes identified through Trendy. Set Size represents the total number of genes identified within each group-direction analysis (i.e., COMBO-“None” genes, COMBO-“Up” genes, etc.). Intersections of genes identified between each analysis are demonstrated by the bar graph. Dots and lines indicate if genes were identified between two or more analyses.

Dynamic changes in gene expression were evaluated for all three groups across all five time points, resulting in the identification of 259, 521, and 268 dynamic genes identified within AAC, COMBO, and INR, respectively (Supplemental File S5). The trends in gene expression after the identification of a breakpoint (“Up”, “Down”, “Stabilized”), or the lack of a breakpoint (“None”), were further examined within each group. For AAC, 40, 29, 101, and 89 genes were identified as “Up”, “Down”, “Stabilized”, or “None”, respectively. For COMBO, 58, 16, 89, and 358 genes were identified as “Up”, “Down”, “Stabilized”, or “None”, respectively. For INR, 15, 77, 143, and 33 genes were identified as “Up”, “Down”, “Stabilized”, or “None”, respectively. Visualization of the number and overlapping of significantly trended genes by group and breakpoint directionality is found in Figure 8. When evaluating gene trend count interactions via the Upset plot, very few overlaps were identified. However, one major overlap identified with respect to the same trend was that of “Stabilized” gene expression within AAC and COMBO, highlighted by seven genes: *CPT1A*, *FCGRT*, *LOC101905776*, *LOC104971307*, *LOC514978*, *PDK4*, and *ZDHHC3*.

Functional enrichments of trended genes found within each group were identified (Supplemental File S6). The total number of enriched GO terms and pathways for gene trends within each group are found in Table 3. For AAC “Up” genes, enrichments were primarily related to the metabolism and translation of RNA, innate immunity, and neutrophil degranulation. For AAC “Down” genes, enrichments were primarily related to amino acid and carbohydrate metabolism, DNA repair and cell cycle, cell membrane signaling and reception, and fatty acid transport. For AAC “Stabilized” genes, enrichments were primarily related to innate immunity, lipid/fatty acid metabolism, phagocytosis, antigen presentation via MHC class I, and ATPase activity. No significant enrichments were identified for AAC “None” genes. For COMBO “Up” genes, enrichments were related to ubiquitin-protein transferase activity, negative regulation of smooth muscle cell differentiation, and negative regulation of miRNA transcription by RNA polymerase II. For the COMBO “Down” genes, enrichments were primarily related to DNA binding and repair mechanisms and cell microtubule activity. No significant enrichments were identified for COMBO “Stabilized” genes. For COMBO “None” genes, enrichments were primarily related to B-cell receptor signaling, interleukin receptor signaling, and mitochondrial activity. For INR “Up” genes, enrichments were primarily related to DNA synthesis and cell cycle, cellular response to stress and external stimuli, heat shock protein production and binding, interleukin 17 signaling, response to interleukin 4, antigen processing and presentation, and mitochondrial translation. No significant enrichments were identified for INR “Down” genes. For INR “Stabilized” genes, enrichments were primarily related to cholesterol and steroid biosynthesis, NF-kappa B signaling, B-cell receptor signaling, inflammasomes, and AMP binding. For INR “None” genes, enrichments were primarily related to innate immunity, cellular response to stress and external stimuli, heat shock protein production and binding, DNA synthesis and cell cycle, autophagy and antigen presentation, neutrophil degranulation, T-, leukocyte, and dendritic cell chemotaxis, and post-translational modification.

## 4. Discussion

Trace mineral supplementation is performed via indirect methods, such as pasture fertilization, or direct methods, such as mineral licks, oral drenches, boluses, and/or parenteral injections. However, factors such as season, individual animal preferences and behavioral patterns, mineral palatability, and delivery methods influence mineral intake, often resulting in over- or underconsumption within the herd [51]. While free-choice minerals are commonly provided to grazing cattle, they do not guarantee optimal intake. As such, energy fortification and protein supplementation are often recommended to ensure proper trace element intake, albeit with potential increases in production costs [52]. To mitigate costs, producers may reduce supplementation frequency, although limited research exists on its impact on performance and physiological parameters. Therefore, the present study serves to investigate this area by evaluating the effects of three trace element supplementation protocols on liver and serum mineral concentrations and whole blood gene expression dynamics in high-risk stocker cattle. Through this research, our goal is to elucidate the potential immunomodulatory and metabolic shifts associated with these strategies over time.

Previous research has been performed with cattle in an effort to identify immunological changes associated with supplied minerals and subsequent biological concentrations [53,54,55,56,57,58,59,60]. Importantly, our work differs from the aforementioned studies in that we leveraged both liver and serum mineral concentrations while comprehensively targeting systemic gene (mRNA) expression in a time-course manner; our approach may provide improved insights to up- and downstream organ systems in relation to mineral supplement protocols instead of from targeted tissue types such as liver or muscle [61]. Moreover, our work utilizes commercial high-risk stocker cattle to capture information related to management programs routinely enrolled in post-weaned cattle production systems. However, several limitations exist in this study. First, our design lacks a control group, and therefore the findings should not be interpreted for effect but for comparison. Additionally, background information related to prior management and genetics for these animals is unknown. While this is a common feature of high-risk cattle management, the association of mineral supplement protocols with prior exposures, such as vaccination, health events, potential progeny differences, and maternal conditioning, is unknown in this study and warrants dedicated research [56,62,63,64,65,66]. Finally, interactions between the gastrointestinal and/or respiratory microbial communities and trace element absorption, with accompanying immunological alterations, were not accounted for in this study and are an important topic in cattle health and management research [67,68,69].

### 4.1. Liver and Serum Mineral Concentrations

Liver and serum cobalt (Co) and copper (Cu) concentrations increased significantly over the first 28 days across all three supplementation groups. This observed increase may suggest efficient bioavailability and accumulation of Co and Cu, indicating the metabolic utilization of these elements within the liver [21]. The lack of significant differences between these groups in the first 28 days indicates that the type of supplementation (organic or inorganic) did not significantly influence Co and Cu accumulation [21,63]. However, liver concentrations of Cu, which may more accurately represent bioavailability, demonstrated a more distinct response compared to serum concentrations [63].

First, Cu concentrations were significantly higher across all three groups at day 60 compared to day 28, suggesting a linear increase over time in terms of hepatic absorption, regardless of supplement regimen. And second, the COMBO group displayed significantly higher copper concentrations compared to the AAC group on day 60. This finding suggests potential synergistic effects between the supplemented trace elements or differential metabolic pathways influenced by the combined supplementation, as seen by differences in the “Down” trending genes and enrichments between the AAC and COMBO groups. Moreover, the positive linear associations observed between liver and serum Co and Cu concentrations suggest coordinated regulation or similar metabolic pathways governing the homeostasis of these trace elements within the body. These findings underscore the importance of considering systemic mineral dynamics and interrelations when assessing the effects of supplementation.

The lack of significant differences in manganese (Mn) and zinc (Zn) concentrations between groups at any time point or within each group over time indicates that the chosen supplementation regimens did not significantly affect the accumulation of these minerals in the liver or serum. While research suggests that Mn and Zn supplement enhances performance (i.e., weight gain, dietary efficiency) and anabolic processes in commercial beef cattle, the source of these elements may not be as important as the general availability within forages [57,70,71,72,73,74,75]. However, notable limitations of this study to comprehensively address the relative effects of Mn and Zn supplementation on transient liver and serum accumulation, in addition to dynamic gene expression, are related to the lack of a negative control group and high-frequency sampling closer to the time point of arrival. It is possible that key biological features influenced by Zn or Mn were missed because they occur early on once cattle begin consuming or are administered these products. Future research directions focusing on addressing these research gaps and investigating the effect of differing geographical locations and production stages in North American beef production systems are highly warranted.

### 4.2. Whole Blood Gene Expression Dynamics

When evaluating sample dissimilarity through PCA, global gene expression patterns appeared to be greatly influenced, specifically by AAC and COMBO supplements, while INR effects were far more variable. Moreover, the same genes, *ADAT1*, *GLRX3*, *LGALS3*, *LOC786987*, *MEI1*, *MTCP1*, *PIK3AP1*, *PRDM5*, *PRPF19*, *SYN1*, *TMEM132B*, and *U2SURP*, were drivers of gene expression patterns (PCs), which possessed significant correlations with the growth rates, supplement allocations, and serum and liver mineral concentrations of these cattle. Of particular interest in immunity and inflammatory signaling, previous research has implicated the importance of these genes with cell survival, inflammatory regulation, and lymphocyte activity [76,77,78,79,80,81,82,83,84,85,86,87]. Moreover, when evaluating trend-wise gene expression over time, consistent “Stabilized” gene expression patterns were found between the AAC and COMBO groups. These shared genes have been identified as important molecules associated with lipid metabolism, glucocorticoid responsiveness, and immunological development and signaling in cattle [88,89,90,91,92]. Furthermore, through a pairwise comparison of groups at each timepoint, minimal differences were observed between the AAC and COMBO groups. While this is relatively expected, considering the only difference between these groups was the administration of the trace mineral drench product (Profusion) upon arrival, more transient effects of the drench product may have been observed if sampling had been performed more frequently (e.g., hourly, daily after administration). These effects may play a critical role in modulating immunity in a more acute fashion and warrant further research. 

Differential expression analysis revealed significant alterations in gene expression profiles between the AAC and INR groups, particularly evident on days 13, 28, and 45. On day 13, differences seen between the AAC and INR groups were primarily related to cell surface activity, transmembrane receptor signaling, ATPase activity, neutrophil degranulation, lymphocyte activation, MCH class I antigen presentation, and IL-4, -7, and -10 cellular activity. The upregulation of genes associated with B- and T-cell activation and proliferation, accompanied by these interleukin-linked gene expression patterns, suggests a potential enhancement of adaptive immune response and homeostasis in cattle receiving AAC supplementation in the first two weeks following facility arrival, when compared to INR supplementation, possibly through dendritic cell-dependent activity [93,94,95,96,97,98]. Conversely, decreased expression of genes related to neutrophil degranulation and platelet activation in AAC compared to INR may reflect alterations in inflammatory and hemostatic processes or a shift in immunological tendencies (i.e., innate versus adaptive immunity reliance) between these two groups.

By day 28, a reduction in the number of DEGs was observed; however, significant enrichment of functional enrichments related to nervous system development, immune responses, and cellular differentiation persisted between AAC and INR cattle. The downregulation of genes in AAC cattle compared to INR cattle involved in B-cell receptor signaling and osteoclast differentiation may suggest a delay in the response around lymphocytic proliferation and physiological development that was comparatively observed earlier (D13) in AAC cattle, albeit through different genes and cellular mechanisms.

On day 45, differentially expressed genes and accompanying enrichments identified between AAC and INR cattle were primarily related to immunological regulation, lipid metabolism, and cytokine signaling. Notably, decreased expression of genes in AAC cattle when compared to INR cattle involved in interferon signaling and bile acid metabolism may suggest potential long-term modulation of inflammatory and metabolic pathways in response to organically sourced trace mineral supplementation [99,100]. Furthermore, the persistent upregulation of genes related to lymphocytic activation underscores the potential immunomodulatory effects of organically sourced trace minerals compared to inorganically sourced trace minerals.

## 5. Conclusions

This study aids in providing a more comprehensive understanding of the complex interactions between trace element supplementation, mineral metabolism, and gene expression dynamics, specifically focused on the immune system. Further investigations are warranted to elucidate the specific molecular pathways and regulatory networks underlying the observed effects, potentially driving precision-based supplementation strategies and therapeutic interventions targeting trace element deficiencies or imbalances in high-risk settings. Additionally, future studies may explore additional time points and cross-system (cow-calf to stocker; stocker to feedlot) assessments to further delineate the temporal dynamics of mineral accumulation and immunological changes. Additionally, the future integration of multi-omics approaches, including incorporating genetic, metabolomic, and metagenomic sequencing data, may provide a more comprehensive understanding of the systemic effects of trace element supplementation.

## Figures and Tables

**Table 1:** Nutritional composition of the organic (AAC, COMBO) and inorganic (INR) diets supplemented.

Item	INR	AAC
**Composition, as-fed basis**		
Cracked corn, %	47.15	47.15
Cottonseed meal, %	19.78	19.78
Cottonseed hulls, %	17.99	17.99
Soybean hull pellet, %	12.89	12.79
Macromineral mix ^1^, %	2.16	2.21
Inorganic trace mix ^2^, %	0.051	0
Organic trace mix ^3^, %	0	0.09
**Nutritional profile ^4^, dry matter basis**		
Dry matter, %	87.8	88
Net energy for maintenance, Mcal/kg	1.56	1.5
Net energy for gain, Mcal/kg	0.97	0.91
Total digestible nutrients, %	65.83	65.53
Acid detergent fiber, %	26.9	27.23
Neutral detergent fiber, %	36.07	34.73
Crude protein, %	16.93	15.7
Ca, %	0.95	0.64
P, %	0.56	0.49
Mg, %	0.31	0.26
K, %	1.02	0.93
Na, %	0.18	0.11
S, %	0.19	0.18
Co, ppm	1.1	1.5
Cu, ppm	21	18.7
Fe, ppm	155	141
Mn, ppm	44.7	40.7
Se, ppm	0.32	0.29
Zn, ppm	77.3	73

^1^ Customized blend of minerals, vitamins, and feed additives (Ware Milling, Houston, MS, USA), which contained sodium monensin (Rumensin: Elanco Animal Health, Greenfield, IN, USA) at 120 g/ton. ^2^ Customized blend of trace minerals (Ware Milling, Houston, MS, USA), which contained Co, Cu, Mn, and Zn as sulfate sources. ^3^ Availa 4 (Zinpro Corporation, Eden Prairie, MN, USA), which contained (dry matter basis) 5.15% Zn from 1:1 Zn and amino acid (AA) complex, 2.86% Mn from 1:1 Mn and AA complex, 1.80% Cu from 1:1 Cu and AA complex, and 0.18% Co from Co glucoheptonate. ^4^ Based on wet chemistry procedures by a commercial laboratory (ServiTech Inc., Dodge City, KS, USA). Calculations for net energy maintenance and gain used equations proposed by the NASEM [20].

**Table 2:** Feed and trace element intake across all three treatment cohorts.

Item	AAC	COMBO	INR	SEM	*p*-Value
Feed intake, kg/day	10.5	10.0	10.0	0.4	0.49
Co, mg/day	11.6 ^b^	15.0 ^a^	14.9 ^a^	0.5	<0.01
Cu, mg/day	220.9 ^a^	186.1 ^b^	186.8 ^b^	7.8	0.004
Mn, mg/day	470.3 ^a^	406.5 ^b^	405.1 ^b^	16.2	0.01
Zn, mg/day	813.2 ^a^	729.0 ^b^	726.6 ^b^	28.7	0.06

Means with different superscripts differ significantly (*p* ≤ 0.05).

**Table 3:** Total number of significant functional enrichments (Gene Ontology (GO), pathways (KEGG and Reactome)) identified by dynamically expressed genes within each group (AAC, COMBO, and INR).

Number of Enriched GO Terms				
	Up	Down	Stabilized	None
**AAC**	34	35	20	0
**COMBO**	3	25	0	15
**INR**	84	0	15	47
**Number of Enriched KEGG and Reactome Pathway**				
	**Up**	**Down**	**Stabilized**	**None**
**AAC**	27	21	12	0
**COMBO**	0	4	0	11
**INR**	31	0	9	39

## Data Availability

The data presented in this study are openly available in the National Center for Biotechnology Information Gene Expression Omnibus (NCBI-GEO), under the accession number GSE225397.

## Supplementary Materials

The following supporting information can be downloaded at: https://www.mdpi.com/article/10.3390/ani14152186/s1, File S1: Liver and serum mineral concentrations from all disease-free cattle; File S2: Metadata for all sequenced healthy cattle samples; File S3: Differentially expressed genes identified between groups through glmmSeq (FDR < 0.05) and edgeR glmQLF testing (FDR < 0.10); File S4: Functional enrichments identified from DEGs discovered through glmmSeq and edgeR testing (FDR < 0.05); File S5: Trend-wise expression of significant genes by trace element supplement group; File S6: Functionally enriched GO terms and pathways for gene trends by supplement group (FDR < 0.05).
